# Freeze-Dried Softisan^®^ 649-Based Lipid Nanoparticles for Enhanced Skin Delivery of Cyclosporine A

**DOI:** 10.3390/nano10050986

**Published:** 2020-05-21

**Authors:** Maria Inês Silva, Ana Isabel Barbosa, Sofia A. Costa Lima, Paulo Costa, Tiago Torres, Salette Reis

**Affiliations:** 1 LAQV, REQUIMTE, Departamento de Ciências Químicas, Faculdade de Farmácia, Universidade do Porto, Rua de Jorge Viterbo Ferreira, 228, 4050-313 Porto, Portugal; ines_afonso_15@hotmail.com (M.I.S.); anabarbosa.cc90@gmail.com (A.I.B.); shreis@ff.up.pt (S.R.); 2 UCIBIO, REQUIMTE, MedTech, Departamento de Ciências do Medicamento, Faculdade de Farmácia, Universidade do Porto, Rua de Jorge Viterbo Ferreira, 228, 4050-313 Porto, Portugal; pccosta@ff.up.pt; 3 Serviço de Dermatologia do Centro Hospitalar e Universitário do Porto, Instituto de Ciências Biomédicas de Abel Salazar, Universidade do Porto, Rua D. Manuel II, s/n, 4099-001 Porto, Portugal; tiagotorres2002@hotmail.com

**Keywords:** cellular uptake, Franz-cell permeation, keratinocytes, pig ear skin, pseudoplastic properties, solid lipid nanoparticles

## Abstract

Inflammatory skin diseases, including psoriasis and atopic dermatitis, affect around one quarter to one third of the world population. Systemic cyclosporine A, an immunosuppressant agent, is included in the current therapeutic armamentarium of these diseases. Despite being highly effective, it is associated with several side effects, and its topical administration is limited by its high molecular weight and poor water solubility. To overcome these limitations, cyclosporine A was incorporated into solid lipid nanoparticles obtained from Softisan^®^ 649, a commonly used cosmetic ingredient, aiming to develop a vehicle for application to the skin. The nanoparticles presented sizes of around 200 nm, low polydispersity, negative surface charge, and stability when stored for 8 weeks at room temperature or 4 °C. An effective incorporation of 88% of cyclosporine A within the nanoparticles was observed, without affecting its morphology. After the freeze-drying process, the Softisan^®^ 649-based nanoparticles formed an oleogel. Skin permeation studies using pig ear as a model revealed low permeation of the applied cyclosporine A in the freeze-dried form of the nanoparticles in relation to free drug and the freshly prepared nanoparticles. About 1.0 mg of cyclosporine A was delivered to the skin with reduced transdermal permeation. These results confirm local delivery of cyclosporine A, indicating its promising topical administration.

## 1. Introduction

Severe inflammatory skin diseases, such as psoriasis and atopic dermatitis, are difficult to control with only topical therapy [1]. In most cases, the use of systemic agents, including immunosuppressants (corticosteroids, cyclosporine, methotrexate, azathioprine) or biologic agents, is needed to effectively control the skin disease. However, oral or parental administration of these drugs is associated with several non-specific interactions, variation of plasma levels, and rapid elimination. This results in the need for multiple administrations, risk of side effects, the possibility of repeated doses, gastrointestinal disorders, and higher dose amounts in order to achieve a therapeutic response [2].

Despite some risks associated with the use of nanotechnology, these drug delivery systems have been shown to increase the effectiveness of the drug, limiting unwanted side effects [3]. In the past few years, nanoparticles have given a new purpose to drugs that presented problems in terms of undesirable side effects, poor water solubility and consequently low bioavailability [4,5]. Solid lipid nanoparticles are one type of the designed drug carriers that can help to overcome some drug-related problems, and they have been extensively reviewed [6,7]. These systems are composed of a lipid that is solid at room temperature and stabilized with a surfactant layer, presenting the possibility to incorporate different drugs and prevent its degradation by controlled release [8].

Solid lipid nanoparticles (SLN) have been reported as possible formulations for cutaneous application, especially taking advantage of the interaction with the skin’s lipids for better drug penetration [9,10,11,12,13,14]. The use of these systems can be a solution to avoid the toxicity of prolonged use of potent immunosuppressive agents, such as cyclosporine A, in the treatment of severe inflammatory skin diseases.

**Figure 1:**
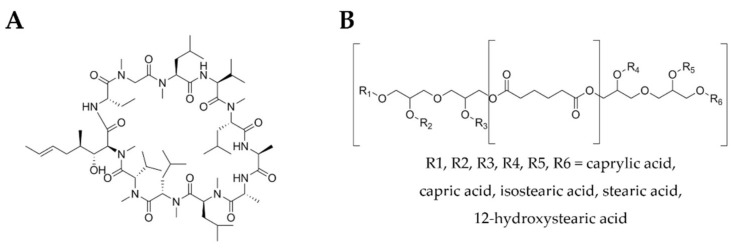
Structures of CsA (**A**) and Softisan^®^ 649 (**B**).

Cyclosporine A (CsA) (Figure 1A) is a widely used immunosuppressive drug that was first used to prevent transplant rejection and later approved by FDA for the treatment of psoriasis [15,16]. This compound is a calcineurin inhibitor that acts selectively on T cells, interfering in the transcription of interleukin-2, which is crucial for full activation of T-cell pathway [17]. CsA has been demonstrated to be highly effective, in the treatment of several inflammatory skin disses, such as atopic dermatitis, psoriasis, pyoderma gangrenosum and chronic idiopathic urticaria, with a rapid onset of action [15]. Despite its great therapeutic potential, CsA-associated side effects are dose-dependent and are closely related to the duration of the applied treatment [18,19]. Hence, topical application represents a solution to overcome this drawback. Given the physicochemical characteristics of CsA, namely its large molecular weight (1202 Da) [20,21] and high lipophilicity (Log P 2.92) [22,23], the skin permeation is a challenge.

To address this problem and to avoid systemic toxicity, many efforts have been made towards research on lipid nanoparticles for topical delivery of CsA. Lopes and co-workers reported monoolein as a solution to enhance the skin penetration of CsA, with reduced transdermal delivery and low systemic effects [24]. Later, Varia et al., tested different lipid matrices to incorporate CsA with high entrapment efficiency percentages (83–97%), reporting a sustained drug release of 7.95% or 41.12% at the end of 20 h, depending on the type of matrix [25]. Kim and coworkers observed in an in vivo murine model the decrease in T helper 2 cell-related cytokines, suggesting that SLN are effective CsA carriers to be applied in allergy-related skin disorders [26]. Guada and colleagues also provided some information on different and stable SLN combinations for oral administration of CsA [27], as well as the study for a controlled CsA release profile and low side effects [28,29,30]. Other types of nanosystems, such as polymeric nanoparticles or micelles, were also used to entrap and deliver CsA by different routes [20,31,32,33,34,35]. Recent topical strategies for CsA delivery focus on the design of PLGA-based nanocapsules to treat severe atopic dermatitis [36] and the preparation of liposomal carriers as ultraflexible systems to assist the topical absorption of CsA [37]. Recently, our research group disclosed the potential of SLN in improved dermal delivery of CsA in relation to nanostructured lipid carriers [38]. SLN of Lipocire DM/ Pluronic F-127 retained CsA in the skin layers with reduced permeation to avoid systemic side effects.

Softisan^®^ 649 (Figure 1B), a lanolin substitute widely used in the cosmetic industry, is an option as a solid lipid to produce SLN. It is based on pure vegetable and synthetic-based raw materials it has no antioxidants, preservatives, residual solvents and paraffins, pesticide residues and allergenic potential. Compared to lanolin, Softisan^®^ 649 is non-occlusive, has a superior adhesion to skin and mucosae, low water content (<0.25%), high water-binding capacity, and good film-forming properties [39]. Softisan^®^ 649 was first proposed as a vehicle for patch testing in 1986 [40], and its use as a reliable lanolin substitute was supported a few years later [41]. Besides its common use in baby care and lip products, protective creams and sun care, Softisan^®^ 649 has also been reported in the literature in antibacterial ointments [42,43], as a semi-solid lipophilic vehicle for oral capsules [44], and for anti-infective coatings for medical implants [45]. Given the clinical interest in delivering CsA through the skin and the potential of cosmetic ingredients in skin research, the SLN obtained with Softisan^®^ 649 were explored in the present work as skin delivery systems for CsA.

## 2. Materials and Methods

### 2.1. Materials

Softisan^®^ 649 was purchased from IOI Oleo GmbH (Witten, Germany), tween 80^®^, cyclosporine A and coumarin 6 were obtained from Sigma-Aldrich (St Louis, MO, USA). All other chemicals and solvents were of analytical grade acquired from Sigma-Aldrich (St Louis, MO, USA) unless stated otherwise. Aqueous solutions were prepared with double-deionized water (Arium Pro, Sartorius AG, Göttingen, Germany), which possesses conductivity values <0.1 µS cm^−1^. For cell culture, fetal bovine serum (FBS), penicillin-streptomycin (10,000 U/mL) mixture, Dulbecco’s Modified Eagle’s Medium (DMEM), Hank’s Balanced Salt Solution (HBSS) and trypsin-EDTA 0.25% were acquired from Gibco^®^ (Invitrogen Corporation, Massachusetts, USA). HaCaT human keratinocyte cell line and L929 fibroblast cell line were purchased from Cell Lines Service (CLS, Eppelheim, Germany).

### 2.2. Preparation of Solid Lipid Nanoparticles

CsA-loaded SLN and unloaded SLN were prepared using the hot ultrasonication method. The lipid phase composed of Softisan^®^ 649 (150 mg), tween 80^®^ (50 mg) and CsA (15 mg) was heated to 65 °C in a water bath. After lipid melting 7 mL of water were added to the lipid phase and then homogenized using a probe-sonicator (VCX130, Sonics & Materials, 115 Newtown, CT, USA) with amplitude frequency of 70% during 5 min, in order to obtain a nanoemulsion. Unloaded SLN were prepared usingds a similar method, without the presence of drug. For cellular uptake assays, SLN were loaded with 1.5 mg of coumarin 6 (C6) upon addition to the lipid phase during the preparation procedure.

### 2.3. Characterization of Solid Lipid Nanoparticles

#### 2.3.1. Average Size and Surface Potential Determination

The mean size, polydispersity index (PDI) and zeta potential of the formulations were determined using a ZetaPALS zeta potential analyzer (Brookhaven Instruments Corporation; Holtsville, NY, USA) [38]. Samples where diluted in double deionized water (1:100), which is the same aqueous phase used to prepare the nanoparticles prior each determination. In particle size measurements, 6 runs of 2 min were performed using Dynamic Light Scattering technique at 20 °C for each assay by the multimodal analysis of ZetaPALS Particle Sizing Software. Zeta potential of the nanoparticles was determined by the Electrophoretic Light Scattering technique with an electrode operating at a scattering angle of 90° at 20 °C. For each assay, 6 runs of 10 cycles were performed.

#### 2.3.2. Morphology Assessment

The morphology of the nanoparticles was observed by Transmission Electron Microscopy (Jeol JEM-1400; JEOL, Ltd., Tokyo, Japan). In each measurement, one drop (ca. 20 µL) of each sample was placed on a copper grid for 1 min. Then, 10 µL of 0.75% (*w*/*v*) uranyl acetate were added as contrast agent and left 30 sec in contact. The excess was removed with filter paper and the grid was exposed at the accelerating voltage of 60 kV for analysis and images capture.

#### 2.3.3. Quantification of the Entrapment Efficiency and Drug Loading

Entrapment efficiency (EE) of CsA in SLN was determined by UV spectrophotometry. A dilution of SLN formulations in double-deionized water was centrifuged (Allegra^®^ X-15R, Beckman Coulter, California, USA) through centrifugal filter units (Amicon^®^ Ultra Centrifugal Filters, Ultracel—50 KDa, Darmstadt, Germany) at 3500 rpm, 20 °C, during 10 min or until complete separation between the SLN retained in the filter unit and the aqueous phase corresponding to the filtrate with unentrapped CsA. The filter unit was then turned over to a tube and centrifuged again at 3500 rpm, 20 °C for 10 min to recover the nanoparticles. The collected nanoparticles were then destroyed with absolute ethanol to release the entrapped CsA. Another step of centrifugation enabled to pellet the lipids and the CsA in the supernatant was quantified by UV–vis spectrophotometry (Jasco V-660 Spectrophotometer, Piscataway, NJ, USA) at 202 nm. Considering the drug initially added to the SLN formulation it was possible to determine the amount of drug incorporated in the SLN, and thus the entrapment efficiency, using the following equation:

(1) $$EE\left(\%\right)=\frac{Entrapped\;drug}{Total\;initial\;drug\;amount}\times 100$$

and the drug loading (DL) by:

(2) $$DL\left(\%\right)=\frac{Entrapped\;drug}{Total\;formulation\;mass}\times 100$$

#### 2.3.4. Freeze-Drying

In the current work, the nanoparticles were frozen overnight at −80 °C (Deep Freezer, GFL^®^, Burgwedel, Germany). After this procedure, the nanoparticles were lyophilized using a freeze drier (LyoQuest −85 plus v.407, Telstar^®^ Life Science Solutions, Terrassa, Spain) for 72 h at −80 °C under 0.40 mbar of pressure.

#### 2.3.5. Fourier-Infrared Spectroscopy Evaluation

The freeze-dried nanoformulations with and without CsA, the Softisan^®^ 649 and CsA were evaluated using a FTIR Spectrophotometer (FrontierTM, PerkinElmer; Santa Clara, CA, USA) equipped with a diamond crystal. The samples were transferred directly into the ATR compartment, and the result was obtained by combining the 16 scans. A background run (to remove the background noise of the instrument) was carried out as a negative control. Spectra were recorded between 4000 and 600 cm^−1^ with spectral resolution of 4 cm^−1^.

#### 2.3.6. Storage Stability Studies

To assess the storage stability of the formulations through time, regular measurements of size, PDI, zeta potential and drug content were performed over a period of 8 weeks. Formulations were stored in sealed glass vials protected from light at 4 °C and at 25 °C to compare the results.

### 2.4. Rheological Properties

The rheological properties were evaluated for the freeze-dried samples of unloaded and CsA-loaded nanoformulations were analyzed on a rheometer (Malvern Kinexus Lab+; Malvern Instruments; Worcestershire, UK) using a shear rate table method (0.1 to 100.0 s^−1^, 10 samples per decade). The analysis was conducted with a plate–plate configuration (geometry PU20 SR4367) with a 1 mm gap at 25 °C (Peltier Plate Cartridge). The rheological properties were studied by continuous shear operation, which was performed to evaluate the shear stress (Pa) as a function of shear rate (s^−1^). The data were collected using the rSpace software^®^ (Kinexus 1.75: PSS0211-17).

### 2.5. Cellular Studies

#### 2.5.1. Cell Culture Conditions

Fibroblasts (L929 cell line) and keratinocytes (HaCaT cell line) were cultured in DMEM supplemented with 10% FBS (*v*/*v*) and 1% penicillin/streptomycin (*v*/*v*). Cells were maintained in a 37 °C and 5% CO_2_ atmosphere (Unitherm CO_2_ Incubator 3503 Uniequip; Planegg, Germany). When reaching 90% confluence, L929 cells were physically detached from the cell culture flask using a scrapper (Nunc^TM^ Cell Screppers, Thermofisher Scientific; Waltham, MA, USA) and HaCaT Cells were chemically detached using trypsin-EDTA 0.25% (*w*/*v*).

#### 2.5.2. Cell Viability Assays

The cytotoxicity assays of the SLN were conducted using 3-(4,5-Dimethylthiazol-2-yl)-2,5-diphenyltetrazolium bromide (MTT) assay. Briefly, L929 and HaCaT were incubated with different concentrations of the SLN at 37 °C for 24 h, at a density of 5.0 × 10^4^ cells per well in a 96-well plate. Cells were treated with 1% (*v*/*v*) triton X-100 as positive control for cytotoxicity. Then, the cells were incubated with MTT solution (0.5 mg mL^−1^) for 2 h at 37 °C. At the end of the incubation, the MTT solution in each well was replaced by 100 µL of DMSO to dissolve the formazan-containing crystals. The plate was shaken for 30 s at room temperature, prior measuring the absorbance of each well at 570 nm and 630 nm (for background subtraction) in a Synergy^TM^ HT Multimode plate reader (BioTek^®^ Instruments Inc., Winooski, VT, USA). The percentage of cell viability was compared to control wells treated with only culture medium by the ratio of corrected absorbance measured for the tested conditions and the untreated cells. All experiments were performed in triplicate.

#### 2.5.3. Cell Uptake Assays

For studying the uptake process of the SLN by HaCaT cells, the cells were seeded in a 24-well plate at a concentration of 2 × 105 cells per well. Cells were incubated with C6-labeled SLN at different concentrations in DMEM at different time intervals. Before flow cytometer analysis in a BD Accuri C6 (BD Biosciences, Erembodegem, Belgium), the cells were washed twice with HBSS, recovered with trypsin-EDTA 0.25% (*w*/*v*), and resuspended in 200 µL of HBSS. Dead cells were excluded with propidium iodide staining by adding 2 µL from a 1 mg mL^−1^ (in water) working solution. Trypan blue at 1 mg mL^−1^ was added to quench the C6 signal from the non-internalized SLN that were only adsorbed on the cell surface. At least 10,000 events were recorded for each sample.

### 2.6. In Vitro Skin Permeation Assay

The skin permeation was tested with CsA-loaded SLN using a Franz cell assembly (9 mm unjacketed Franz Diffusion Cell with 5 mL receptor volume, o-ring joint, clear glass, clamp, and stir-bar; PermeGear, Inc., USA). Pig ear skin was used as model barrier. The donor medium consisted of 0.5 mL with 1 mg CsA-loaded SLN, free CsA or 100 mg of freeze-dried CsA-loaded nanoformulation. The receptor medium was 4.7 mL of HEPES buffer (pH 7.4) with 10% ethanol, to allow CsA dissolution. The available diffusion area between chambers was 0.785 cm^2^. The stirring rate and temperature of receptor medium were respectively kept at 400 rpm and 37 °C (IKAMAG^®^, Staufen, Germany). At defined intervals of 1 h, 1 mL aliquots of the receptor medium were collected and immediately replaced with equal volumes of fresh buffer. Each sample was quantified by UV–vis spectrophotometry (Jasco V-660 Spectrophotometer, Piscataway, NJ, USA).

### 2.7. Statistical Analysis

Statistical analysis was performed using GraphPad Prism Software (Version 7 for Windows; GraphPad Software Inc, San Diego, CA, USA).

## 3. Results and Discussion

### 3.1. Physicochemical Characterization of Softisan^®^ 649/Tween^®^ 80-Based Nanoparticles

After using hot ultrasonication method to prepare unloaded and CsA loaded nanoparticles, the physicochemical characterization was obtained, and the results obtained are summarized in Table 1. The formulations presented an average size around 200 nm, which is within the adequate size range for topical delivery [46]. The polydispersity index for the prepared nanoparticles was below 0.2, a representative value of formulations with a narrow range of particle sizes. Table 1 data evidenced zeta potential values for SLN of −15 ± 4 mV, and −22 ± 2 mV upon the incorporation of CsA, indicating the need to verify their colloidal stability during storage. It is well established that when surface values are equal to or higher than |30| mV, the nanoparticles have electrostatic stabilization and low tendency to aggregate [47]. The CsA entrapment efficiency was around 88% (approximately 13 mg in 7 mL of SLN), with a drug loading of approximately 7%.

### 3.2. Morphology Analysis

**Figure 2:**
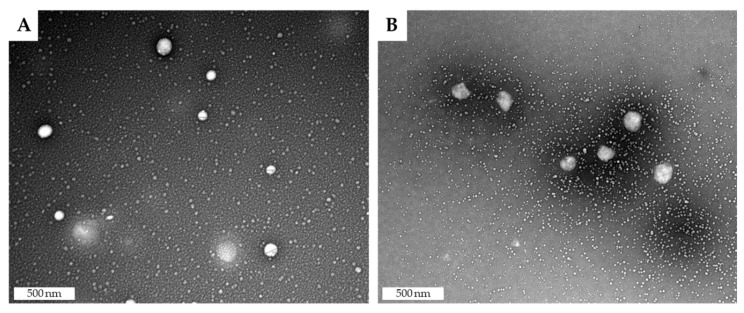
Morphology of (**A**) SLN and (**B**) CsA-SLN.

The morphology of the nanoparticles was evaluated by transmission electron microscopy (TEM). Figure 2 show SLN with spherical appearance, and that entrapment of CsA did not affect the nanoparticle’s morphology.

### 3.3. Fourier-Infrared Spectroscopy Evaluation

**Figure 3:**
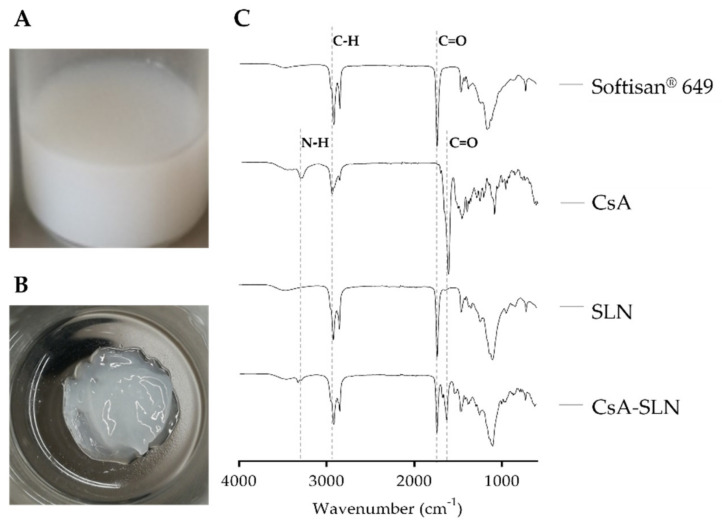
CsA-SLN freshly prepared (**A**) and after freeze-drying process (**B**) and FTIR spectra (**C**) of reference compounds (Softisan^®^ 649 and CsA) and freeze-dried nanoformulations (SLN and CsA-loaded SLN).

The FTIR analysis was performed after freeze-drying nanoparticles to validate the CsA incorporation into the lipid nanoparticles (Figure 3C). The freshly prepared CsA-loaded SLN (Figure 3A) after the freeze-drying process exhibited a gel-like appearance (Figure 3B). The characteristic peaks of the analyzed compounds are detailed in Figure 3C. As represented in the spectra of CsA, the main peaks are related with N–H (3300 cm^−1^) and amide I C=O (1625 cm^−1^) [48]. Softisan^®^ 649 is mainly characterized by alkyl C–H (2920 cm^−1^) and ester C=O (1740 cm^−1^) [49]. As expected, the spectrum of SLN is very similar to the spectrum of Softisan^®^ 649. The combination of characteristic peaks of Softisan^®^ 649 and free CsA appears in the spectrum of CsA loaded nanoparticles, and no new bonds appear, indicating that cyclosporine was successfully incorporated without altering its chemical structure or the structure of the nanoparticles.

### 3.4. Assessment of the Storage Stability

**Figure 4:**
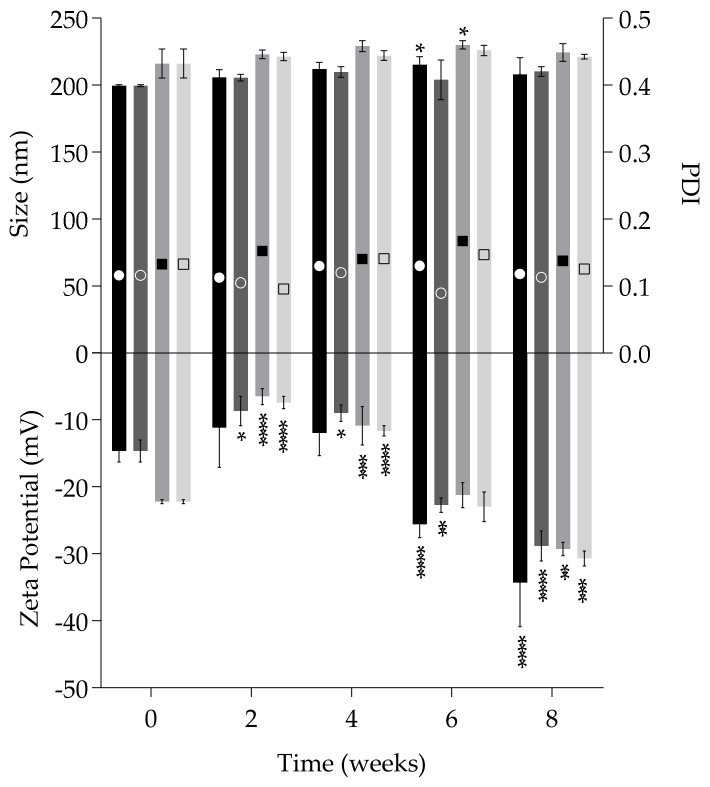
Storage stability of formulations at room temperature and 4 °C over 8 weeks. Size and Zeta Potential data: SLN at room temperature (black bars) and 4 °C (dark grey bars); CsA-loaded SLN at room temperature (grey bars) and 4 °C (light grey bars). PDI data: SLN at room temperature (white circle) and 4 °C (white open circle); CsA-loaded SLN at room temperature (black square) and 4 °C (black open square). Each result represents the mean ± standard deviation for n = 3 samples. * *P* < 0.05; ** *P* < 0.01; *** *P* < 0.001; **** *P* < 0.0001.

Storage stability was evaluated at room temperature and 4 °C in terms of particle size, PDI and zeta potential over 8 weeks. Figure 4 show the size of unloaded and CsA-loaded SLN remain constant during the period of study at both temperatures. In terms of polydispersity values, which are in all cases below 0.2, it is possible to find the same trend suggesting low tendency for aggregation. The zeta potential was the most variable parameter with significant changes due to increasing negative values over time. This variation may be attributed to the multiple structural configurations of Softisan^®^ 649 molecules. Softisan^®^ 649 is a mix of fatty acid esters with a melting point of approximately 35 °C [49]. However, it is submitted to heat (60 °C) to facilitate the CsA dissolution in the production of nanoparticles, which means it will take a longer time to reach a stable conformation and position of the matrix. In fact, with longer periods of time, the more negative the values become, reaching −30 mV in both conditions for all formulations, which indicates higher colloidal stability. Despite these significant changes, the stability does not seem to be affected when considering data from size and PDI.

### 3.5. Cellular Studies

#### 3.5.1. Cell Viability

Biocompatibility of lipid nanoparticles was confirmed as recommended by ISO 10993 on “Biological evaluation of medical devices” using the L929 fibroblasts cell line. For a more representative analysis according to the purpose of the study, the same assay was performed in HaCaT keratinocytes, a skin characteristic cell line. According to ISO 10993-5, if the cell viability is lower than 70% of the control, there is a cytotoxic effect.

**Figure 5:**
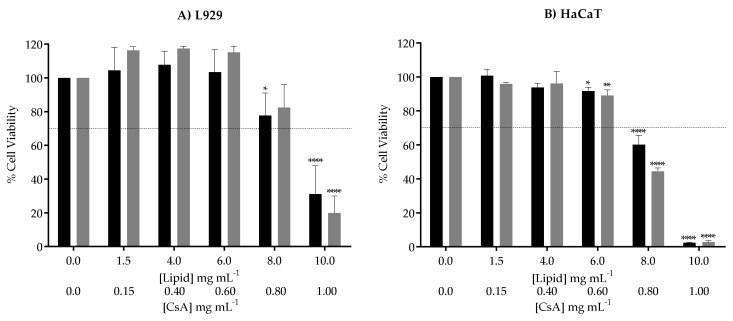
Cellular viability determined by the metabolic activity of (**A**) L929 fibroblasts and (**B**) HaCaT keratinocytes upon 24 h of exposure to both unloaded (black bars) and CsA-loaded SLN (grey bars). The dotted line represents 70% of cell viability as a reference for non-toxic concentrations. Each result represents the mean ± standard deviation for n = 4 replicates of 3 assays. * *P* < 0.05; ** *P* < 0.01; **** *P* < 0.0001.

Analyzing Figure 5, it is possible to observe biocompatibility and safety of SLNs up to concentrations of 6 mg mL^−1^ in lipid for both cell lines, corresponding to a CsA concentration of approximately 0.6 mg mL^−1^. In previous studies performed by the group, free CsA was toxic for L929 when using concentrations higher than 35 µg mL^−1^ and for HaCaT using concentrations higher than 17.5 µg mL^−1^ [38]. For the higher tested lipid concentrations (8 and 10 mg mL^−1^) containing 0.8 and 1 mg mL^−1^ in CsA, respectively, cell viability decreased to values below 70%, indicating a cytotoxic effect that was more evident in keratinocytes (Figure 5B) than in fibroblasts (Figure 5A), when compared to the control cells. Compared to other studies involving SLN, we were able to confirm non-cytotoxicity at higher concentrations (up to 6 mg mL^−1^ in lipid loaded with 0.6 mg mL^−1^ of CsA), revealing the biocompatibility of the designed nanoparticles [26,50].

#### 3.5.2. Softisan^®^ 649/Tween^®^ 80-Based Nanoparticles Internalization by Keratinocytes

**Figure 6:**
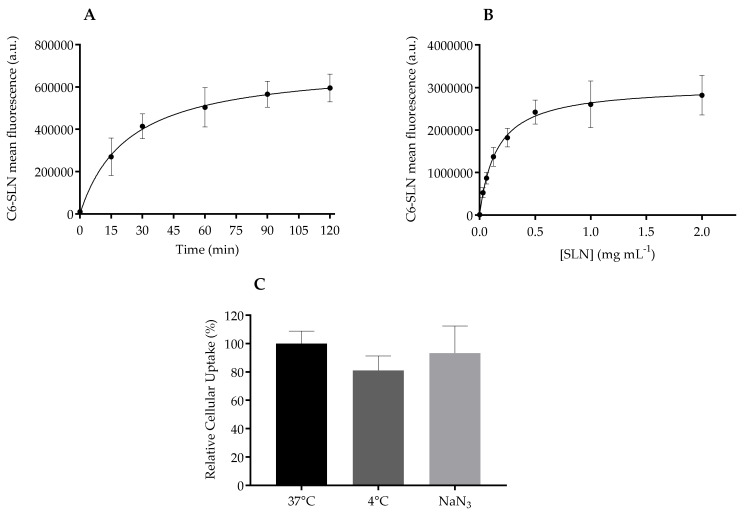
Cellular uptake for C6-loaded SLN in HaCaT cells. (**A**) Effect of incubation time on nanoparticle internalization at 0.05 mg mL^−1^ in lipid; (**B**) Effect of concentration on nanoparticle internalization at 37 °C for 1 h; (**C**) Role of energy in endocytosis of C6-SLN at 0.05 mg mL^−1^ in lipid, in relation to 37 °C cellular C6 fluorescence. Data expressed as mean ± standard deviation (n = 6).

The cellular uptake of C6-loaded SLN was assessed using flow cytometry. According to kinetic analysis (Figure 6A), it is possible to see an increase of mean fluorescence over time, due to an increasing accumulation of nanoparticles within the cells. Flow cytometry data showed a time-dependent accumulation (Figure 6A). The internalization process is fast, since nanoparticle translocation is detected after 15 min of nanoparticle’ incubation and continues to increase until achieving a plateau after 90 min. A concentration-dependent internalization was observed, with a saturation plateau at a low nanoparticle concentration of 0.5 mg mL^−1^ in lipid (Figure 6B).

Overall, the internalization routes for nanoparticles to enter cells can be divided into active and passive pathways. The influence of temperature on cellular uptake is often tested to evaluate the active transport that is reduced under low temperature (4 °C), and thus nanoparticles are internalized via energy-dependent transport (endocytosis) or energy-independent mechanisms (non-endocytic pathways). In this work, HaCaT cells were incubated at two inhibitory conditions of active transport: 4 °C and sodium azide [51]. At 4 °C, energy-dependent uptake and passive diffusion are hampered due to cell membrane rigidity, and in the presence of sodium azide the active transport is blocked due to inhibition of electron transport chain in the mitochondria, thus impairing the active uptake.

Figure 6C shows the relative cellular uptake in percentage, assuming that normal internalization (37 °C) fluorescence of cells in the absence of any inhibitor was 100%. There is no statistical difference between the internalization of the control and the inhibitory conditions, revealing that Softisan^®^ 649/Tween^®^ 80 nanoparticles enter keratinocytes by an energy-independent process. The quick translocation of the designed SLN into HaCaT might be related to its nanometric size, but this feature does not seem to affect the viability of cells as observed with other lipid compositions [52].

The overall data suggests that SLN quickly translocate into the cells, saturating at very low concentrations by an energy-independent process, so these systems will easily and safely interact with keratinocytes in a topical application scenario.

### 3.6. Rheological Properties of Freeze-Dried Nanoformulations

**Figure 7:**
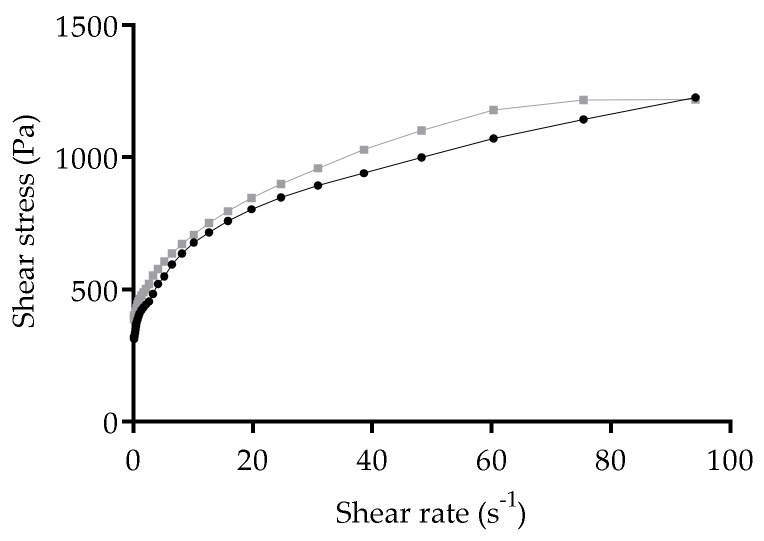
Flow curves for unloaded (black dots) and CsA-loaded nanoformulation (grey squares) regarding shear stress.

At the end of the freeze-drying process, the unloaded and CsA loaded Softisan^®^ 649/Tween^®^ 80-based SLN formed a gel-like matrix. The morphology of freeze-dried samples was not checked, yet the observed nanoparticle structure in the freshly prepared samples may have changed. During the freeze-drying process, the water was removed, and given the composition of the Softisan^®^ 649/Tween^®^ 80 nanoparticles, an oleogel was formed. Since oleogels have also been considered to be a good base for transdermal formulations [53], the freeze-dried Softisan^®^ 649/Tween^®^ 80 nanoformulations were assessed for skin application (Figure 3B), and thus rheological properties were evaluated. The flow curves describing the measurement of shear stress (Pa) with increasing shear rate (s^−1^) are shown in Figure 7. Unloaded and CsA-loaded nanoformulations presented shear thinning behavior: shear stress increases (Figure 7) and shear viscosity decreases with increasing shear rate (data not shown). Pseudoplasticity is important for topical systems, because with increasing shear stress the material will flow better through skin, providing a good skin penetration of the active substances. The presence of CsA in the Softisan^®^ 649 oleogel does not seem to affect its thixotropic properties, due to the similarity in terms of shear stress and viscosity responses.

### 3.7. In Vitro CsA Skin Permeation Studies

**Figure 8:**
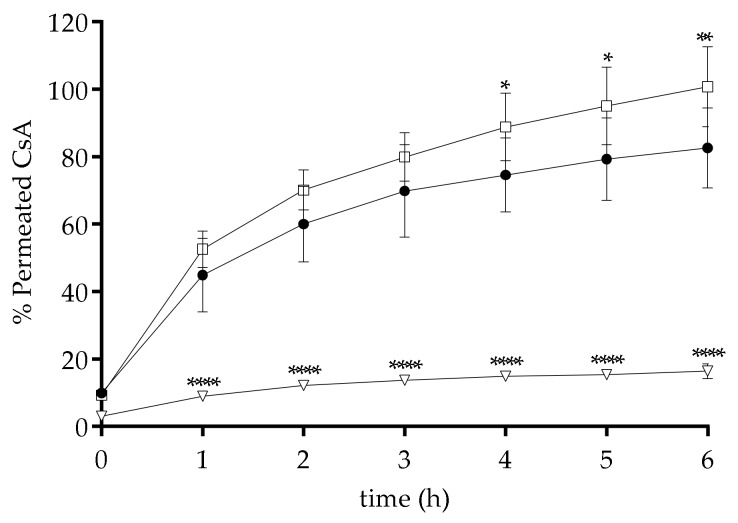
Permeation extent through pig ear skin of free CsA (black circles), CsA-loaded SLN (open squares) and freeze-dried CsA-loaded nanoformulation (open triangles) under skin conditions. Data expressed as mean ± standard deviation of n = 4 replicates for each tested condition. * *P* < 0.05; ** *P* < 0.01; **** *P* < 0.0001.

To evaluate the permeation of CsA through the skin, the Franz cell diffusion assay was applied. Pig ear skin was chosen as model barrier due to its similarity with human skin in terms of morphology and function [54,55]. Since the permeation detection was needed, HEPES buffer (pH 7.4) with 10% ethanol was used to mimic physiological conditions in the basolateral compartment, without compromising CsA solubility. Figure 8 shows the obtained results for the 6 h experiment on skin permeation analysis.

The results reveal an increase in the permeation of CsA upon incorporation in the nanoparticles and a reduced permeation of drug within the oleogel in comparison to the percentage of permeated free drug. The CsA present in the oleogel form of the nanoparticles exhibits constant permeation after the first hour of assay. By the end of 6 h of assay, only 15% of CsA permeated through pig ear skin from the freeze-dried nanoformulations in contrast to the ca. 80% permeation observed with the free CsA. On the other hand, the CsA-loaded nanoparticles present a fast diffusion through the skin and immediate effect, suggesting that Softisan^®^ 649 might act as a penetration enhancer compound to be applied in order to overcome the high molecular weight and lipophilicity of CsA in skin delivery. The main drawback of this permeation profile is the CsA amount, which could reach systemic distribution with the inherent side effects of the compound. Due to the enhanced skin penetration of the chosen lipid when it is in liquid form, CsA-SLN are transported across the full skin, showing a statistically higher permeation than free CsA after 4 h of assay. However, when the CsA-loaded nanoformulations are freeze-dried, the particle acquires an occlusive function, which enables skin penetration, maintaining a local effect. This suggests that freeze-dried Softisan^®^ 649/Tween^®^ 80 nanoformulations might result in a local release profile, limiting the amount of CsA that reaches physiological media, and consequently lowers the undesired systemic side effects of the drug, as shown in previous studies [56].

CsA deposition after 6 h from the freeze-dried Softisan^®^ 649/Tween^®^ 80 nanoformulations exceeded 1.0 mg/cm^2^. This outcome surpasses other literature reports of CsA loaded in nanoparticles for skin delivery using porcine skin [24,33,57,58], most probably given the high CsA content in the freeze-dried nanoformulation (6.6%), and the presence, in the receptor medium, of ethanol to assure skin conditions. The latter may have increased skin permeability by diffusing into the tissue from the dermis, as evidenced by the presence of ca. 15% CsA in the receiver compartment after 6 h. Other effects contributing to this increase in penetration may occur, such as increased diffusive flux into the skin of the oleogel compared to the nanoparticles. Skin delivery of CsA from a monoolein/propylene glycol formulation was around 100 μg/cm^2^. In another study, nano-dispersions of monoolein/ oleic acid containing 0.6% (*w*/*w*) of CsA lead to 61 μg/cm^2^ deposition after 12 h application [24]. Micelles of MPEG-dihexPLA delivered supra-therapeutic amounts of CsA to porcine skin (1.1 ± 0.5 μg/cm^2^) after 1 h of contact [33]. Protamine-based nanosystems deposited ca 100 µg/cm^2^ of CsA upon 24 h of application in 2–3-day-old pigs [57]. Higher deposition values of CsA in the skin (ca. 450 µg/cm^2^) were reported for amorphous nanoparticles loaded with 5% of drug [58] corresponding to almost half of the achieved amount delivered with freeze-dried Softisan^®^ 649/Tween^®^ 80 nanoformulations.

In conclusion, the freeze-dried Softisan^®^ 649/Tween^®^ 80 nanoformulation appears to be a better delivery system for CsA than other nanoparticle-based system reported in literature. It allows delivery and retention of CsA in the skin, avoiding its transdermal permeation and potential systemic exposure to the drug in vivo. Nevertheless, a validation in diseased human skin is necessary, as the barrier function in severe inflammatory skin diseases can be highly modified. Softisan^®^ 649/Tween^®^ 80 nanoformulation has the advantage of being a repurposing of a safe cosmetic ingredient into an innovative formulation in drug delivery, with good biocompatibility and skin deposition of the loaded drug.

## 4. Conclusions

In this work, Softisan^®^ 649/Tween^®^ 80-based nanoformulations loaded with CsA are proposed as a solution to allow the penetration of CsA into the skin barrier. To the best of our knowledge, Softisan^®^ 649 has not been reported in the production of CsA nanoparticles, despite being a very well-known cosmetic ingredient with several topical applications. The characteristics of this lipid are very interesting with the objective of topical administration of CsA, acting as an enhancer for the penetration of drugs already used in the clinic, but which reveal problems related to high molecular weight or high lipophilicity, such as CsA.

The nanoparticles were successfully produced and characterized, presenting suitable size for topical delivery, low polydispersity index values and negative surface charges with incorporation of CsA in the nanoparticle structure reaching about 7% of drug loading capacity. Given that 88% of CsA was successfully entrapped into the designed SLN, it was possible to increase the drug aqueous solubility by 157-fold, when considering the experimental value of 0.012 mg mL^−1^ [59]. Biocompatibility studies revealed safe usable concentrations up to 0.6 mg mL^−1^ of CsA, equivalent to 6 mg mL^−1^ in lipid. Freeze-drying process of the CsA-loaded SLN resulted in an oleogel with pseudoplastic behaviors. Due to the disclosed in vitro controlled permeation profile of freeze-dried CsA-loaded nanoformulation and considering their rheological properties, it is possible to consider this new vehicle for topical administration of CsA, avoiding its systemic side effects. This delivery system presents an advantage over conventional SLN of direct application to the skin, avoiding the mandatory incorporation of colloidal nanoparticles in a vehicle (gel, ointment, cream). We believe that the incorporation of CsA into the SLN based on Softisan^®^ 649 and Tween^®^ 80 associated with the freeze-drying process may bring new strategies for the topical administration of CsA in the management of severe skin diseases.

## Figures and Tables

**Table 1:** Physicochemical characterization of nanoparticles.

	Size (nm)	PDI	ζ-Potential (mV)	EE (%)	DL (%)
SLN	200 ± 4	0.12 ± 0.03	−15 ± 4	-	-
CsA-SLN	216 ± 5	0.11 ± 0.02	−22 ± 2	88 ± 3	6.6 ± 0.2

Data expressed as mean ± standard deviation (n = 6).
